# Recurrent Cutaneous Myxoma of the Eyelid: A Case Report

**DOI:** 10.1155/crop/9241454

**Published:** 2026-04-17

**Authors:** Mariana I. Fonseca, Christian Nieves-Rios, Jorge Billoch, Joseph Campbell

**Affiliations:** ^1^ Department of Medicine, Ponce Health Sciences University, Ponce, Puerto Rico, USA, psm.edu; ^2^ Department of Ophthalmology, School of Medicine, University of Puerto Rico Medical Sciences Campus, San Juan, Puerto Rico, USA, upr.edu; ^3^ Department of Pathology, HRP Labs, Hato Rey, Puerto Rico, USA

**Keywords:** cutaneous, eyelid, myxoma, recurrent

## Abstract

Cutaneous myxomas are rare, benign mesenchymal tumors of the dermis. They may occur in isolation or as part of genetic syndromes and can affect the head and neck regions, yet eyelid involvement is rare. We report a case of a 24‐year‐old male with a 10‐year history of a recurrent right upper eyelid lesion, previously excised five times. Clinical examination revealed a pearly, nodular lesion, and histological findings of dermal fibroplasia and dilated vessels, initially misdiagnosed as a hemangioma. Subsequent recurrence and excisional biopsy showed hypocellular myxoid stroma with embedded spindled cells, confirming the diagnosis of cutaneous myxoma. Systemic evaluation excluded syndromic associations such as cardiac myxoma. The patient remained recurrence‐free at 3 months postoperatively. Early‐onset disease, multiple recurrences, and unusual location emphasize the need for careful clinicopathologic correlation and long‐term follow‐up. Recognition of this rare entity is critical for appropriate management, exclusion of systemic associations, and reduction of recurrence risk. This case highlights that recurrent eyelid lesions, even when previously diagnosed as benign vascular lesions, should prompt reconsideration of rare myxoid tumors.

## 1. Introduction

Myxomas are characterized by sparsely cellular lesions composed of stellate or spindle‐shaped cells embedded within an abundant myxoid extracellular stroma [1, 2]. Although cardiac muscle is most affected, myxomas can involve other tissues, including skeletal muscle, bone, skin, the genitourinary and alimentary systems, nasal sinuses, and, in rare instances, the orbit and ocular adnexa [3]. Eyelid involvement is exceptionally rare, with only a limited number of cases reported in the literature.

Cutaneous myxomas, also referred to as superficial angiomyxomas, typically present as slow‐growing, painless, solitary or multiple nodules on the trunk, extremities, or head and neck [2, 3]. These lesions usually measure less than 5.0 cm in diameter and may occur in isolation or as part of genetic syndromes such as Carney complex and its related variants [2, 3]. They generally affect individuals between 20 and 40 years of age [2, 3], with variations in reported rates of occurrence between males and females [2, 4, 5].

Clinically, eyelid cutaneous myxomas can resemble other lesions, such as dermoid cyst and papilloma, making diagnosis challenging [3]. Identification of a skin myxoma warrants consideration of systemic associations, including cardiac myxomas, spotty cutaneous hyperpigmentation, and endocrine abnormalities [2, 6]. We present a case of isolated recurrent cutaneous myxoma of the eyelid in a young patient, with initial misdiagnosis of hemangioma.

## 2. Case Report

A 24‐year‐old male initially presented with a 10‐year history of a recurrent lesion involving the right upper eyelid. The lesion had been previously excised five times at an outside institution, with the most recent excision approximately 4 years prior to presentation. No pathology reports were available from preceding excisions. The patient complained of irritation but denied pain, bleeding, discharge, or ulceration. Review of systems was remarkable for a blue nevus on the right arm.

**Figure 1:**
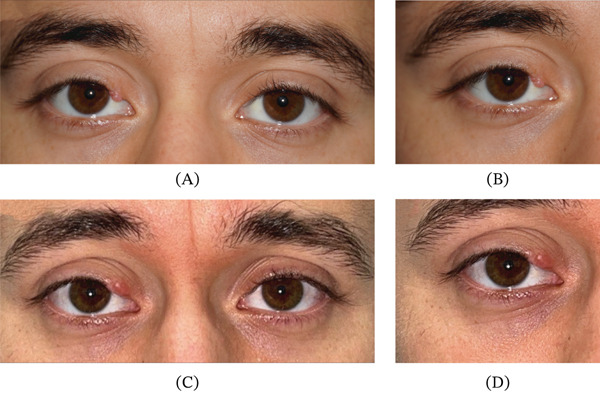
External photographs at initial presentation and after recurrence. (A, B) Images show a pearly, nodular lesion of the right upper eyelid with madarosis and minimal telangiectasia. (C, D) The 5‐year follow‐up images demonstrate recurrence of the lesion.

On examination, visual acuity was 20/20 in both eyes. Intraocular pressures were normal bilaterally with normal pupillary response and complete extraocular movements. External examination revealed an 8.0 × 4.0 mm pearly, nodular lesion of the right upper eyelid with madarosis and minimal telangiectasia (Figure 1A,B). There was no evidence of proptosis or ptosis, and the anterior segment examination was unremarkable. A tissue biopsy demonstrated dermal fibroplasia with dilated vessels, suggestive of a hemangioma.

**Figure 2:**
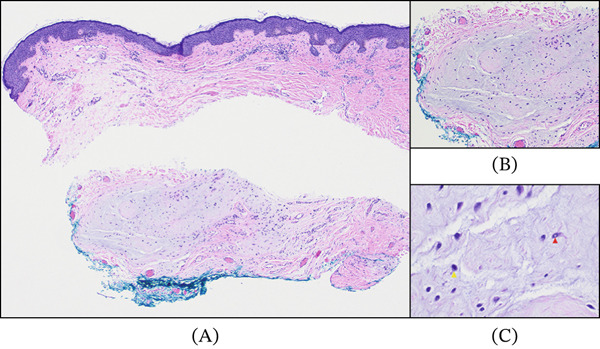
Histologic appearance of the cutaneous myxoma. (A) The low‐power image shows ill‐defined dermal‐based lobulated nodules. Higher magnification highlights hypocellular myxoid stroma (wispy bluish to grayish material) with scant embedded spindled cells with (B) bland nuclei and intranuclear vacuoles (red arrowhead), and (C) scattered mast cells (yellow arrowhead).

He was lost to follow‐up and returned 5 years later due to recurrence of the lesion that had been noticeable for about 18 months. Examination demonstrated a 6.0 × 4.0 mm flesh‐colored, nodular lesion with associated madarosis and no visible telangiectasia (Figure 1C,D). An excisional biopsy of the new lesion was performed, and histopathological analysis revealed findings consistent with a cutaneous myxoma with negative surgical margins (Figure 2A, B, and C). Systemic evaluation included cardiac echocardiography and endocrine assessment, which were unremarkable. There was no evidence of recurrence or complication at the 3‐month follow‐up visit. The patient was scheduled for yearly follow‐ups to monitor for signs of recurrence.

In summary, our patient developed his first lesion at 14 years of age. Between the ages of 14 and 20, he underwent five excisions, yet with an unknown pathological diagnosis. At age 24, he presented at our institution and was initially diagnosed with hemangioma. Five years later, a recurrence was observed, and a repeat biopsy confirmed findings of isolated cutaneous myxoma.

## 3. Discussion

Cutaneous myxoma is a benign tumor that develops in the superficial or deep dermis [6]. It is most frequently diagnosed in middle‐aged individuals [2, 3], and is especially rare in younger patients [3, 6]. Skin myxomas typically affect the trunk, extremities, head, and neck regions, and can rarely present as an isolated eyelid lesion [2, 6, 7]. To our knowledge, only eight well‐documented cases of isolated eyelid cutaneous myxoma have been published in the English literature [3, 6–12], of which only half of them described recurrence.

Sporadic cutaneous myxomas are frequently solitary, whereas patients with syndromes such as Carney complex generally have multiple lesions [6]. Grossly, they can present as soft, fluctuant papules, polypoids, or nodules that may elevate the overlying skin [2]. These lesions may be moderately well circumscribed or poorly defined and generally measure between 1.0 and 5.0 cm, but can range from 0.5 to 14.0 cm [2]. On cut section, they may appear gray to white and have a glistening, translucent, and gelatinous consistency, occasionally containing areas of hemorrhage [2].

Definitive diagnosis of cutaneous myxoma relies primarily on histopathologic evaluation. Characteristic findings include abundant myxoid stroma, mild to moderate cellularity composed of spindle‐shaped to stellate cells with minimal or no cytologic atypia, a delicate arborizing vascular network, and absence of nuclear pleomorphism, hyperchromasia, or mitotic activity [2, 5]. A reported distinguishing feature is the presence of stromal neutrophils in the absence of associated necrosis or ulceration [7, 8]. Immunohistochemical findings in cutaneous myxoma show considerable variability across published reports, particularly for markers such as vimentin, CD34, S‐100 protein, smooth muscle actin, muscle‐specific actin, and factor XIIIa, creating additional diagnostic challenges [1–3, 5, 7, 8]. Stoieva and O′Donnell reported a case of recurrent eyelid cutaneous myxoma in a pediatric patient that exhibited striking histological variability across multiple recurrences [6]. Our case further extends the literature by describing a patient with a decade‐long course of repeated clinical misdiagnoses due to varied histological findings that initially resembled a hemangioma, and emphasizes how incomplete sampling or early biopsies in younger patients may miss classic myxoid features.

The differential diagnosis for cutaneous myxoma includes several myxoid lesions. Clinicopathologic correlation, including architectural features, vascular patterns, and cellular details, helps distinguish among these entities. Myxoid neurofibroma features prominent wavy collagen bundles and sharply angulated nuclei, along with myxoid change, yet lacks the delicate arborizing vascular network and stromal neutrophils typical of cutaneous myxoma [2].

Nerve sheath myxoma is predominantly dermal, displays well‐defined, multinodular, or lobulated architecture with nests of plump epithelioid or spindle cells, without the prominent vasculature of cutaneous myxoma [2, 4]. Myxoid dermatofibrosarcoma protuberans shows more uniform and bland spindle cells in a storiform pattern with very infiltrative borders and higher overall cellularity [13]. Lastly, focal cutaneous mucinosis consists of dermal mucin pools with minimal cellularity and lacking delineation, lobulated growth, prominent vasculature, and stromal neutrophils [13].

Cutaneous myxomas may present as an initial syndromic manifestation; therefore, patients should be evaluated for systemic associations, particularly cardiac myxomas, due to their high associated morbidity and mortality. In our patient, the presence of a blue nevus on the arm raised concern for a possible syndromic relationship; however, no comorbidities were identified. Management of cutaneous myxomas usually involves a shave biopsy or wide local excision. Although these tumors are benign and without metastatic risk, these lesions have a rate of local recurrence between 30% and 40%, occurring as early as 2 months or as late as 20 years after initial removal [1, 2, 6, 7]. This case highlights the potential for cutaneous myxoma to manifest as a long‐standing, recurrent lesion in young patients, even in the absence of syndromic features. A limitation of this report is the lack of histopathologic records from previous excisions and no long‐term follow‐up after the last excision. Although follow‐up is currently limited to 3 months, long‐term yearly surveillance for recurrent periocular myxomas is warranted, given their indolent but persistent nature and reports of late recurrence, particularly in cutaneous myxomas. Early clinicopathologic correlation, including repeat biopsy if initial findings are atypical, is essential to avoid diagnostic delay. Ophthalmologists should consider cutaneous myxoma in recurrent eyelid lesions even when prior histopathology suggests more common diagnoses.

## Funding

No funding was received for this manuscript.

## Ethics Statement

This study adheres to the tenets of the Declaration of Helsinki, and in accordance with the Health Insurance Portability and Accountability Act (HIPAA) regulations.

## Consent

Consent to publish the case report was obtained from the patient. This study adheres to the tenets of the Declaration of Helsinki, and in accordance with the Health Insurance Portability and Accountability Act (HIPAA) regulations.

## Conflicts of Interest

The authors declare no conflicts of interest.

## Data Availability

The data that support the findings of this study are available on request from the corresponding author. The data are not publicly available due to privacy or ethical restrictions.
